# Investigating the interaction between human immunodeficiency virus, nutrition, and disability: A cross-sectional observational study

**DOI:** 10.4102/phcfm.v10i1.1663

**Published:** 2018-06-20

**Authors:** Hellen Myezwa, Jill Hanass-Hancock, Nikolas Pautz

**Affiliations:** 1 Department of Physiotherapy, University of the Witwatersrand, South Africa; 2 HIV-Prevention Research Unit, School of Health Science, University of KwaZulu-Natal, South Africa

## Abstract

**Background:**

The average lifespan of people with human immunodeficiency virus (HIV) has increased because of the enhanced access to anti-retroviral treatment. This increased longevity has led to a heightened focus on the comorbidities which may arise, allowing a clearer understanding of the contextual, personal, psychological and functional problems and their interrelations. Disability (functional limitations) and insufficient nutritional intake may interact cyclically with HIV and/or acquired immunodeficiency syndrome (AIDS); however, no research to date has investigated this interaction.

**Aims:**

The objective of this article was to report on the nutritional outcomes using albumin and body mass index outcomes as a subset of a larger study among adults living with HIV and/or AIDS.

**Setting:**

This study was conducted at a large HIV clinic based in an urban area in Johannesburg, South Africa, which provides HIV treatment and support to over 6000 persons with HIV and TB. This clinic is part of a large public health regional hospital where extensive HIV research is undertaken.

**Methods:**

This study was a cross-sectional observational study. The sample composed of 278 participants between 18 and 65 years of age and had been on highly active antiretroviral therapy (HAART) for more than six months. Statistical analyses were performed using the Statistical Package for the Social Sciences.

**Results:**

The results indicated that albumin level had significant inverse associations with functional limitations and physical health symptoms. Women were significantly more likely to have lower nutritional levels. A logistic regression analysis suggested that gender and physical health symptoms were the primary predictors of albumin levels.

**Conclusion:**

The findings presented in this article can be applied to HIV and/or AIDS treatment programmes, such as HAART. It re-emphasises the importance of providing individuals on anti-retroviral therapy with affordable and adequate nutrition, education on the importance of nutritional intake and the benefits of potentially adopting supplement programmes. As females seem to be more adversely affected by low nutritional levels, with the findings showing an increased likelihood of developing physical health symptoms, focus also needs to be given to cultural or social factors that impact nutritional intake in women.

## Introduction

The level of impact that the human immunodeficiency virus and/or acquired immunodeficiency syndrome (HIV and/or AIDS) has on people’s lives and on community development is overwhelming.^1,2,3^ HIV remains one of the greatest public health concerns contributing to mortality and, increasingly, morbidity in the Southern African region.^4,5^ The disease and its social impact can also have devastating effects on livelihoods.^6,7^ Anti-retroviral therapy (ARV) slows down the progression of the virus but does not eliminate it. The new South African National Strategic Plan on HIV, TB and sexually transmitted infections (2017–2022) outlines the need to undertake a health wellness approach to managing HIV as people living with HIV (PLHIV) increasingly live longer.^8^ The life span of PLHIV is now close to those who are without the disease.^9^

Managing HIV is increasingly being approached from the perspective of chronic disease models of care including a biopsychosocial approach.^10,11^ Much research has been undertaken to inform the biomedical, behavioural and psychological elements of HIV.^6,12,13^ Elements related to disability and functional limitation which impact wellness across the lifespan have recently received attention with a better understanding of functional limitations among PLHIV.^5^

Earlier studies showed high levels of impairments and functional limitations in the muscular skeletal, sensory (including pain) and mental health domain.^14,15^ Several studies including two systematic reviews also showed that functional limitations and/or disability are potentially experienced by a large number of PLHIV in Africa including those on anti-retroviral therapy (ART).^4,16^ More recent studies are illustrating the complexity of the intersection of HIV health-related quality of life, functional limitations and disability, as well as health and wellness.^5,16,17^ For instance a recent study with people living with HIV revealed that functional limitations/disability are associated to depressive symptoms in particular in women living with HIV.^6,18^ Studies indicate that people living with HIV who also experienced functional limitation score not only worse for depressive symptom but also have works health related and ART adherence outcomes.^6,15,18^ Additionally, these participants also had worse livelihood outcomes and scored low on food security scales.^6^ These difficulties calls for models of care that address new health-related challenges and comorbidities of living with long-term HIV.^19,20^

**FIGURE 1:**
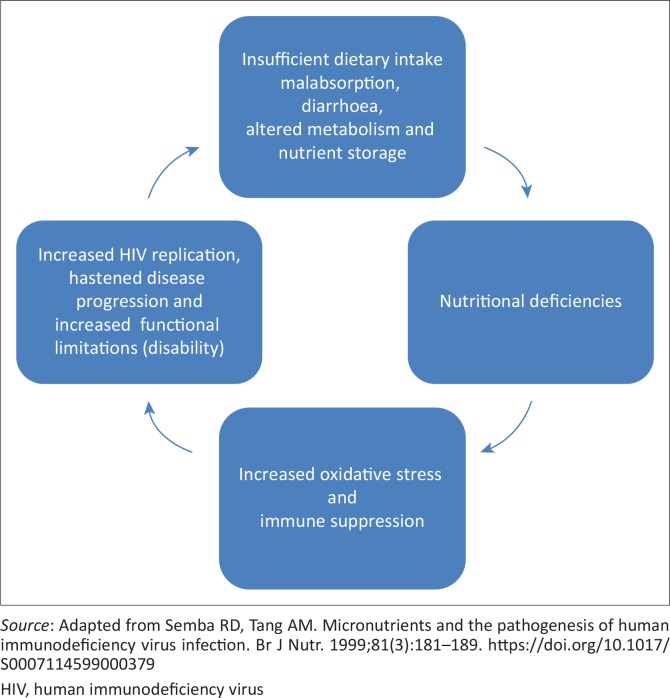
Cyclical nature of human immunodeficiency virus and nutrition.

The recognition that social determinants play a large role in the emergence and management of HIV has also gained greater understanding and contributes to new models of care.^21^ The study undertaken by Hanass-Hancock et al.^6^ revealed that livelihood measures (aggregate and individual) were associated with a number of socio-demographic factors such as age and gender. In this study, food, security and aggregate livelihood were significantly worse for women and older individuals than for men or younger people.^6^ Considering that food security was one of the factors associated with health, livelihood outcomes and functional limitations, it is of interest to understand how outcomes relate to nutrition. Nutrition is a closely related health outcome and an important element in the management of HIV treatment and care. Associations have been found between livelihood, capitals and health indicators such as HIV-related health, symptoms, body mass index (BMI), activity limitations and depressive symptoms.^6,18^ The relationship between nutrition and HIV has been studied widely; Figure 1^22^ illustrates how insufficient dietary intake can influence disease progression and in turn increase morbidity, which could increase the presence of functional limitations.

As Figure 1^22^ illustrates, nutritional status is adversely influenced by infections and malabsorption, which may over time increase the risk of functional limitations.^23^ Episodes of illness and the experience of functional limitations may impact a family’s ability to provide an income at the expense of depleting household assets,^24^ exacerbating the effects of a poor dietary intake. A low BMI is associated with poor clinical outcomes (low CD4 and opportunistic infections), even after ART initiation.^25^ Therefore, adequate nutrition is seen as important to support recovery from opportunistic infections and reverse weight loss, as well as important for embarking on ARV treatment as malnutrition directly increases the body’s susceptibility to HIV infection.^26^ Additionally, it has been argued that poor nutrition may affect a patient’s adherence to ART by depriving the individual of the energy to travel to collect their prescribed medication or because of medications toxic effects.^13^ How nutrition relates to other HIV-outcomes, such as mental health outcomes, functional limitations or livelihood has not been described in greater detail.

Adequate nutrition is needed for many aspects of cognitive functioning,^27^ and poor nutritional status has been increasingly recognised as a critical determinant of poor mental health.^28^ Multiple analyses based on data from both resource-limited and resource-rich settings suggest an association between food insecurity and poor mental health among people living without^29^ or with HIV.^30^ Several cross-sectional studies have also described the association between food insecurity resultant nutritional status and depression among PLHIV,^31,32^ suggesting that nutritional intake may be a risk factor for the mental well-being of individuals. For instance, depression has been reported as being twofold higher in individuals living with HIV,^33^ increasing to two and a half fold when the CD4 cell count is below 200 mm^2^.^34^ This is especially true in the era prior to widely available ART. However, results are inconclusive post-ART roll-out, with some studies showing positive change in people on long-term ART.^7^

Nutritional status is measured in some studies by using anthropometric and biochemical measures (such as haemoglobin), and albumin, which is a family of globular proteins.^35^ Low albumin may be an independent risk factor for poor cognitive status and mental health problems, such as dementia and depression.^36,37,38^ Previous research has found that low albumin was independently associated with poor cognitive performance in older adults, while low BMI was not.^39^ These results suggest that while an individual may not be underweight, the nutritional value of the food they are eating may not be sufficient. In one study involving an HIV positive population, low albumin levels were found to be moderately associated with BMI, as well as having significantly lower levels in households with low food security.^35^ Additionally, findings suggest that a relationship may exist between albumin levels and HIV progression.^35,40^

A review of the literature shows that nutrition outcomes in HIV cohorts under ART treatments have been reported in terms of dietary intake, BMI measurement, body composition, weight history, serum levels of proteins, lipids, micronutrients, immunological parameters and clinical assessment which includes the assessment of illnesses, malabsorption, medication and illicit drug use.^41^ However, no literature could be found that examined nutritional outcomes and functional limitations except an indirect link to the loss of muscle mass which in turn may be debilitating.^42^

It is well established that nutritional requirements need to be part of managing the treatment of PLHIV.^43^ In one arm of this study, cohort albumin levels, which can be considered as a quantitative proxy for nutritional status outcomes,^44^ were determined for a subset of this larger study. The overall aim of this study was to determine the outcomes of HIV management in terms of functional limitations and/or disability, medical, ART adherence, and nutritional and livelihood outcomes. The objective of this article is to report on the nutritional outcomes using albumin and BMI outcomes as a subset of a larger study among adults living with HIV.

## Methods

### Description of the study settings

This study is one of the three studies (HIV Live Study). This study was conducted at a large HIV clinic based in an urban area in Johannesburg, South Africa, which provides HIV treatment and support to over 6000 persons with HIV and TB. This clinic is part of a large public health regional hospital where extensive HIV research is undertaken. The results presented in this article are a subset of a larger study where the results have been published elsewhere.^6,16,18^

### Study design

This study used a cross-sectional observational design. A comprehensive literature review informed the items for this cross-sectional survey questionnaire for the overall study referred to as the HIV live study.

### Participants

The sample size was calculated at 1050 with Stata 12.1 with a one-sample comparison of proportions (one-sample size computation), a 90% power and two-sided test, and an alpha level of 5% with a hypothesised 55% of the population with disability. For the albumin testing, every patient was invited and only those who consented were tested.

Participants at the clinic were approached consecutively as they queued for their medication and routine checks or to consult a medical officer. Participants were between 18 and 65 years of age and had been on HAART for more than 6 months. Individuals with any acute infection, opportunistic condition or pregnancy were excluded.

### Data collection

Data were collected over a period of 12 months. The questionnaire content was piloted using the first 10 participants who were part of the study and the findings were used to train the interviewers. Difficulties and any mistakes were identified, and reasons for inaccurate capture of information were identified; interviewers comprising the principal investigator and two research assistants, a PhD student and a counsellor discussed the sections for a common understanding.

The questionnaire included demographic details, livelihood, food security indicators and medical history including adherence and CD4 counts. It also included the disability measures (WHODAS 2.0), depressive symptoms (CESD10), HIV-related health outcomes, adherence measures^45^ and measures of exposure to interventions.^6^ We also included nutritional measure using albumin tests^46^ and BMI. A blood sample was obtained from those participants who permitted it. The total albumin protein content in the plasma was tested. Table 1^6,12,45,46,47,48,49^ outlines the tools and their psychometric properties.^6,18^

**TABLE 1:** Outline of the psychometric properties of instruments used in this study.

Elements measured	Tool	Variables	Source, reliability and validity data
Socio-demographic	Socio-demographic questionnaire	Age, gender and marital status (other factors are indicated in the livelihoods section)	-
Livelihood	HIV-Live livelihood capitals (self-developed from the literature)	Education, financial capital, including source of income, physical and natural capital including housing, source of income, water and sanitation, social capital and food security	Livelihood: 0.728 Physical and natural capital: 0.699 Social capital: 0.908 Food security: 0.790^6^
Medical history	Medical symptoms questionnaire	Confusion, memory loss, breathlessness, fatigue, diarrhoea, nausea, stomach pain, headache, change in taste, skin itching/changes, muscular pain, heartburn, sore mouth, vomiting, fever and kidney stones. Included in this section were the patients’ weight, height and CD4 count	Duran S, Spire B, Raffi F, et al.^47^
Physical and functional health	WHODAS 2.0 (5-point Likert scale)	Mobility (standing and walking), self-care (washing and getting dressed), participation (involvement in life situations), cognition (learning concentration and memory), getting along (dealing with people and maintaining friendship) and life activities (work and education)	Internal consistency reliability was 0.94 (Cronbach’s alpha). It showed moderate convergent validity with EQ5D and RAND-12 (0.41–0.76)^48^
Mental health	CESD-10 (4-point Likert scale)	Ten questions prompting status regarding depression, the level of bothering, how fearful, hopeful, happy or lonely one is, and the effort required to get going.	CESD-10 showed good internal consistency reliability with the original CESD-20 (α = 0.88)^12,49^
Adherence	CASE adherence index	Three unique adherence questions combined to form a composite score	CASE adherence index showed a strong correlation with the 3-day self-reported adherence (ROC curve > 0.86, *p* < 0.001).^45^
Nutritional outcomes	Albumin	Albumin serum extracted from blood sample	Human serum albumin (HSA) is the most abundant circulating protein in the body representing about 50% of the overall protein content in the body.^46^

Note: Please see the full reference list of the article, Myezwa H, Hanass-Hancock J, Pautz N. Investigating the interaction between human immunodeficiency virus, nutrition, and disability: A cross-sectional observational study. Afr J Prm Health Care Fam Med. 2018;10(1), a1663. https://doi.org/10.4102/phcfm.v10i1.1663, for more information.

WHODAS, World Health Organization Disability Assessment Schedule 2.0; CESD, Center for Epidemiologic Studies Depression Scale.

### Framework and tools

The tools which were used to collect the data for this study, as well as the reliability and validity of the items, are detailed in Table 1.

### Data analysis

The sample was first described using descriptive statistics. The normal range for albumin is between 35 and 50.^50^ This was in line with the clinical manager living with the HIV virus at the clinic who advised the use of a cut off of 40 based on his clinical experience of patients possibly at risk. Those whose albumin scores were below 40 (at risk of inadequate nutrition) were placed in one group and those were above 40 (adequate nutrition) in a second group. Differences between the two albumin groups were tested using independent sample *t*-tests for continuous data and Fisher’s exact test for categorical data, including the subcategories of the WHODAS 2.0 questionnaire. Associations were tested using a Pearson’s correlation analysis. According to the recommendation,^51^ independent variables with a *p*-value of 0.25 or less were entered stepwise into a multivariate model. All statistical analyses were performed using SPSS^®^ (V23) with an alpha of 0.05 for all tests, unless otherwise stated. Post hoc power tests using G*Power (V3.13) revealed that the power of the logistic regression analysis was 0.96.

## Ethical considerations

Ethical approval was obtained from the Human Research Ethics Committee at the Faculty of Health Sciences, University of the Witwatersrand, Johannesburg, South Africa M131187, and written informed consent from all the participants was obtained.

## Results

As shown in Table 2, the sample of people was gender-biased, with 72% of the sample being women. Most participants fell between the age of 35 and 45 years. Of the sample population, 64% were earning an income and hence had some form of employment. Overall the sample acquired good adherence and good mental health outcomes, while experiencing a number of functional limitations and HIV-related health symptoms. A large number of participants (50%) had experienced shocks (where shocks were measured by the number of adverse events such as the loss of a loved one) within the last 6 months.

**TABLE 2:** Descriptive statistics of sample.

Characteristics	Whole cohort (*n* = 278)				Cohort with albumin < 40 g/dL (*n* = 108)				Cohort with albumin > 40 g/dL (*n* = 170)				*p*
	*n*	%	mean	± SD	*n*	%	mean	± SD	*n*	%	mean	± SD	
Age	-	-	42.48	8.26	-	-	42.76	7.17	-	-	42.3	8.91	0.637
**Gender**													**0.019**
Men	76	27.2	-	-	21	19.4	-	-	55	32.4	-	-	-
Women	202	72.4	-	-	87	80.6	-	-	115	67.6	-	-	-
**Marital status**													0.777
Never married, single	139	50	-	-	56	51.9	-	-	83	48.8	-	-	-
Currently married	72	25.9	-	-	27	25.0	-	-	45	26.5	-	-	-
Divorced, separated or widow	54	19.42	-	-	21	19.44	-	-	33	19.41	-	-	-
Cohabiting	13	4.68	-	-	4	3.7	-	-	9	5.3	-	-	-
**Education status**													0.756
No formal schooling	7	2.5	-	-	2	1.9	-	-	5	2.9	-	-	-
Some primary or primary completed	43	15.47	-	-	17	15.74	-	-	26	15.29	-	-	-
High school	203	73.02	-	-	82	75.93	-	-	121	71.18	-	-	-
Post-secondary	24	8.99	-	-	6	5.6	-	-	18	10.59	-	-	-
Refused or do not know	1	0.4	-	-	1	0.9	-	-	-	-	-	-	-
**Source of income**													0.064
Earned income	178	64.03	-	-	73	67.59	-	-	105	61.77	-	-	-
Gifts	3	1.1	-	-	2	1.9	-	-	1	0.6	-	-	-
Disability grant	3	1.1	-	-	2	1.9	-	-	1	0.6	-	-	-
Other grants	25	9	-	-	10	9.3	-	-	15	8.9	-	-	-
Other income	33	11.8	-	-	15	13.9	-	-	18	10.7	-	-	-
Do not know	35	12.5	-	-	6	5.6	-	-	29	17.1	-	-	-
Income mean	-	-	5030.76	4703.41	-	-	4823.33	4030.32	-	-	5160.92	5088.96	0.582
WHODAS weighted score (mean out of 36)	-	-	5.56	6.38	-	-	6.05	7.16	-	-	5.25	5.83	0.312
CESD -10 score (mean out of 40)	-	-	12.25	7.04	-	-	13.14	7.13	-	-	11.69	6.94	0.094
Adherence score (mean out of 16)	-	-	15	2.04	-	-	14.99	1.92	-	-	15.01	2.12	0.952
Years living with HIV	-	-	7.5	3.91	-	-	7.8	4.17	-	-	7.31	3.75	0.310
Health symptoms (mean out of 16)	-	-	5.48	3.43	-	-	6.11	3.39	-	-	5.09	3.41	**0.015**
BMI (cut-off 25)	-	-	25.32	5.6	-	-	26.01	5.99	-	-	24.9	5.31	0.107
CD4 count	-	-	371.07	225.23	-	-	356.92	222.76	-	-	380	226.97	0.407
**Exposure to shock**													0.805
Yes	140	50.9	-	-	53	49.5	-	-	87	51.8	-	-	-
No	135	49.1	-	-	54	50.5	-	-	81	48.2	-	-	-
**Type of shock**													
Crime	36	13.1	-	-	14	13.1	-	-	22	13.1	-	-	-
Illness and/or injury	82	29.8	-	-	29	27.1	-	-	53	31.5	-	-	-
Death of loved ones	87	31.6	-	-	35	32.7	-	-	52	31	-	-	-
Political unrest	15	5.5	-	-	4	3.7	-	-	11	6.5	-	-	-
Hazards	7	2.5	-	-	2	1.9	-	-	5	2.9	-	-	-
Albumin (g/L)	41.17	4.97	-	-	36.31	3.95	-	-	44.27	2.42	-	-	**0.001**

BMI, body mass index; WHODAS, World Health Organization Disability Assessment Schedule; SD, standard deviation; g/L, gram per litre.

Note: Bold values are significant at 0.05 alpha.

The albumin groups were comparable in terms of their age, years on ART and CD4 count but not in terms of gender. Significant differences between the albumin groups (apart from the actual albumin levels) were related to gender (where women comprised 80.6% of the <40 albumin group, compared with 67.6% of the >40 albumin group) and HIV-related health symptoms (where the <40 albumin group had on average one more symptom opposed to the >40 albumin group).

**FIGURE 2:**
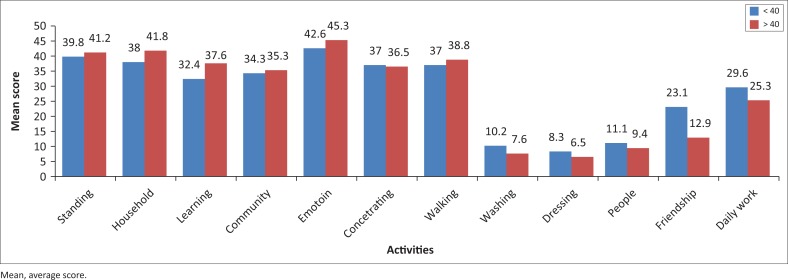
Activity limitations between <40 and >40 albumin count groups.

As shown in Figure 2, slightly higher, but non-significant, levels of limitation were seen in participants with lower nutritional outcomes in washing, dressing, people, friendship and daily work limitation categories. After calculating chi-square tests using Fisher’s exact score, no significant difference among functional limitations in any of the categories based on albumin level categorisation was found.

In the correlation analysis shown in Table 3, a statistically significant weak–moderate negative relationship was evident between albumin levels and the measure of functional limitations and/or disability (*r* = −0.134) and physical health (*r* = −0.18). Additionally, it suggests that as albumin levels increase, the levels of functional limitations and/or disability (WHODAS) and physical health symptoms decrease.

**TABLE 3:** Association between albumin and other variables of interest.

Variables	1	2	3	4	5	6	7	8	9	10	11
Albumin	-	-	-	-	-	-	-	-	-	-	-
Years with HIV	−0.080	-	-	-	-	-	-	-	-	-	-
Age	−0.029	0.127*	-	-	-	-	-	-	-	-	-
Adherence score	0.075	−0.019	0.034	-	-	-	-	-	-	-	-
WHODAS score	−0.134*	0.093	0.157**	−0.081	-	-	-	-	-	-	-
Physical health	−0.180**	0.116	0.074	0.081	0.259**	-	-	-	-	-	-
Shock exposure	−0.025	0.092	0.003	−0.034	0.362**	0.307**	-	-	-	-	-
BMI	−0.079	0.111	0.061	0.007	−0.003	0.032	−0.065	-	-	-	-
Mental health	−0.057	0.080	0.120*	0.002	0.278**	0.417**	0.194**	−0.038	-	-	-
Last CD4 cell count	0.021	0.419**	0.087	−0.041	0.091	0.011	0.088	0.154*	0.174**	-	-
Income	0.029	−0.003	−0.046	0.003	−0.083	−0.197**	−0.079	0.051	−0.101	0.060	-

BMI, body mass index, WHODAS, World Health Organization Disability Assessment Schedule.

* , Significant at the 0.05 level;

** , significant at the 0.01 level.

To further investigate these relationships, a binary logistic regression was undertaken. We used a logistic regression model (Table 4) to investigate the effects of WHODAS 2.0 score, physical health and gender on albumin categorisation. The WHODAS 2.0 score was discarded according to the Wald criterion because of being non-significant (*p* = 0.14). The logistic regression model was statistically significant (χ² [2] = 9.154; *p* = 0.01). The model explained 7.1% (Nagelkerke R²) of the variance in albumin categorisation. Prediction success overall was 60%. This suggests that while gender and physical health as variables have a 60% accuracy of predicting whether an individual has an albumin count of greater than or less than 40, they only explain 7.1% of the total variance that occurs in the albumin categorisation, suggesting other factors play a role in nutritional health.

**TABLE 4:** Logistic regression model.

Variable	*β*	s.e.	*T*	Beta	Sig.	B 95% CI	
						Lower	Upper
Gender	−2.48	1.17	−3.72	−0.22	0.001	−3.79	−1.17
Physical health	−0.185	0.67	−2.15	−0.13	0.033	−0.36	−0.02

s.e., standard error; CI, confidence interval; Sig., significance; *β*, Unstandardized beta coefficient; *T*, critical *t*-value; Beta, Standardized beta coefficient.

Table 4 illustrates that together gender and physical health symptoms have a statistically significant negative relationship with increased albumin levels.

## Discussion

Overall this cohort of patients could be described as attaining good nutritional outcomes as the BMI and albumin levels are predominantly within the reference range (9.7% of the participants had albumin levels of <35). The level of nutrition as measured by albumin in this subgroup was 41.17 g/L, while those who had measures of 40 and below had a mean of 36.31 g/L (SD = 3.95) and those above 40 had a mean of 44.27 g/L (SD = 2.42). This is comparable with the results reported by Stambullian et al.^52^ who reported a mean of 45 g/L for both patients living with AIDS and who are HIV positive, but these results are different from the findings of Santos and Almeida^40^ who found that malnourished individuals had an albumin level of 28.1 g/L. Periodic biochemical parameters are encouraged^52^ in the management of HIV patients. This is done only if the clinical picture for an individual patient indicates a need to do so. Participants who had <40 g/L albumin levels had a lower mean of CD4 count (356.92) compared to those with >40 g/L (380.04). Although this result was not significant, it suggests unsurprisingly that the nutritional level of the participants had some influence over disease progression.

As part of this large cross-sectional study, the disability outcomes of HIV and nutrition management were considered to be important variables in mobility, strength and functional outcomes of patients. Although functional limitations and/or disability emerged originally as a factor associated with levels of albumin, this did not hold when controlling for other factors. However, functional limitations may have an indirect impact, which was not shown in the model. Poor nutrition status may be a predictor of disability even where overt disability is not manifest. A possible explanation could be that low albumin which indicates a lower level of nutrition may influence levels of fatigue and muscle strength. Indirectly this may manifest as disability in terms of lower exercise tolerance and an inability to sustain function. No literature could be found that has sought to assess this relationship although Salomon et al.^41^ suggested that its importance in strength and functional status for the person living with HIV needs attention. Because of the negative impact of nutritional deficiency, nutritional assessments have been proposed and implemented in many HIV management programmes.^42^

Factors associated with nutritional outcomes are important for the holistic management of HIV. Knowing these factors will contribute to a more sensitive and specific programme that addresses the important elements influencing the overall outcome of managing HIV. Factors that correlated with albumin inversely in this study, albeit weakly, were years with HIV, physical health, shock and exposure, BMI, mental health and gender. Further analysis in a regression model revealed significant associations with gender and physical health. It was found that, as albumin levels increase, symptoms of physical health decrease, and the probability of being classified as a male, according to the model, increases. Interestingly, in a related concept to nutritional outcomes, is that being a woman is associated with food insecurity and the occurrence of depression.^28^ Unsurprisingly, the state of physical health was also associated with nutritional outcomes.

There was a moderate and significant inverse correlation between physical health and albumin levels, as shown in the bivariate analysis, suggesting that as albumin levels increase, physical symptoms of ill health decrease. Additionally, from the logistic regression, it is evident that the lower an individual’s physical health, the lower their albumin levels will be. Although the association is not strong, it is statistically significant. This may be because of the relationship between albumin levels and disease progression.^35,40^ Indeed, this finding provides further evidence that albumin is a negative acute-phase reactive protein which is depressed by elevated levels of cytokines, which can occur during chronic inflammation.^35^ This is supported by the fact that the number of years an individual has been living with HIV is inversely, though not significantly, correlated to albumin levels and the fact that there is a linear, but again not significant, correlation between years living with HIV and the number of physical health problems experienced. While individuals with albumin levels of less than 40 had higher, but not statistically significant, scores on the CESD10 questionnaire (13.14) compared to those with an albumin count greater than 40 (11.69), the association between mental health and albumin was weakly inversely associated. Association between mental health and disability has been found in previous studies.^6,18^ Alongside the finding of a significant inverse relationship between WHODAS score and albumin count, it may be hypothesised that nutritional deficiencies may cause, or possible be the effect of, heightened physical deterioration, which in turn may cause an increase in depressive symptoms. Thus, if nutritional needs are met, PLHIV may be less likely to develop mental and physical health symptoms, such as depression, muscle weakness and fatigue.^36^

While only 278 participants were sampled out of a possible 1050, which was a limitation, the power of arguably the most complex statistical test in this research was high. This outcome, in addition to the fact that not many studies that have had as large a sample as this study included albumin level testing among HIV patients, adds to the reliability of the findings.

## Conclusion

This article focuses on investigating the interaction between nutritional levels (albumin) and functional limitations and/or disability in a cohort of people on long-term ART. It was found that there were gender discrepancies in the low- (<40) and high-albumin (>40) groups, with females being significantly more likely to belong to the low-albumin group. Additionally, the low-albumin group had on average a significantly higher count of physical health symptoms compared to participants who had >40 albumin count. No differences were found in individual WHODAS 2.0 categories between the albumin groups. Moderate inverse associations were found between albumin levels, overall WHODAS 2.0 score, gender and physical health symptoms. A linear regression analysis found that, together, gender and physical health symptoms are negatively associated with an increase in albumin levels. Based on the way the variable of gender was coded, this finding can be interpreted as follows: the higher the albumin level, the more likely a participant is to have less symptoms of ill physical health and to be a male.

The findings presented in this article can be applied to HIV and/or AIDS treatment and management programmes outlined in the HIV-planned strategies of prevention treatment care and support. It re-emphasises the importance of providing individuals on ART with affordable adequate nutrients, education on the importance of nutritional intake and the benefits and potentially adopting supplement programmes such as that developed by Cantrell et al.^53^ As females seem to be more adversely affected by low nutritional levels, with the findings showing an increased likelihood of developing physical health symptoms, focus also needs to be on cultural or social factors that impact nutritional intake in women.
